# Management and survival outcomes of patients with lung cancer at a leading radiotherapy centre in Sub-Saharan Africa: a cross-sectional study

**DOI:** 10.3332/ecancer.2025.1924

**Published:** 2025-06-11

**Authors:** Joseph Daniels, Kofi Adesi Kyei, Nduhura Israel

**Affiliations:** 1 National Centre for Radiotherapy, Oncology, and Nuclear Medicine, Korle-Bu Teaching Hospital, PO Box KB 369, Accra, Ghana; 2 Department of Radiography, University of Ghana, PO Box KB 143, Legon, Accra, Ghana; 3 Uganda Cancer Institute, Upper Mulago Hill Road, Box 8754, Kampala, Uganda; a https://orcid.org/0000-0002-1466-150X; b https://orcid.org/0000-0003-3485-5368

**Keywords:** SCLC, NSCLC, lung cancer, smoking history, cancer survival

## Abstract

**Background:**

Lung cancer is a major global health challenge that has a substantial impact on the health and well-being of many individuals. Despite the progress that has been made in its treatment globally, there is a paucity of reliable data on management and survival outcomes in sub-Saharan Africa.

**Aim:**

To describe the clinical profiles, management and survival outcomes of patients with lung cancer.

**Methods:**

This research was a cross-sectional study conducted at a leading radiotherapy centre in Ghana. The study involved adult patients with lung cancer who received treatment over a 10-year period. A consecutive random sampling technique was used to select eligible participants for the study. Relevant data were extracted from patients’ hospital-based medical records. Data were analysed with STATA (version 17). Kaplan–Meier survival analyses were used to estimate overall survival (OS) outcomes. Cox proportional hazards models were used to identify prognostic factors associated with survival outcomes.

**Results:**

In all, there were 118 patients with a male predominance of 53.4%. The mean age was 59.4 years (± 13), with a significant proportion being geriatric (> 60 years) (48.3%). Also, there were more nonsmokers than smokers (57.6% versus 33.9%), with only 6.8% having a positive family history for cancer. Notably, 4.2% had a history of treatment for pulmonary tuberculosis, while ~75% were diagnosed at advanced stages of lung cancer (stage IVA = 48.3% and IVB = 27.1%). Most patients were diagnosed with non-small cell lung cancer (NSCLC) (94.9%). Management was essentially palliative (65.3%), and with the use of systemic therapy (72.3%). Only 3.4% and 1.7% received targeted and immunotherapy, respectively. Patients <60 years had better OS compared with those ≥ 60 years (*p* = 0.771). Similarly, patients with NSCLC had better OS than patients with small cell lung cancer (*p* = 0.001). A good Eastern Cooperative Oncology Group performance status was a predictor of better OS (*p* = 0.004).

**Conclusion:**

The predominance of advanced-stage disease, reliance on palliative care, systemic chemotherapy and disparities in access to advanced therapies highlight significant challenges faced by healthcare providers. Addressing these barriers through targeted interventions, infrastructure investments and policy changes could significantly improve lung cancer outcomes in the region. A focus on early detection, comprehensive diagnostics and equitable access to advanced treatments is essential for enhancing survival rates and quality of life for lung cancer patients in this region.

## Introduction

Lung cancer remains a leading cause of cancer-related morbidity and mortality worldwide, with over 2.5 million new cases and 1.8 million deaths recorded annually [1, 2]. Despite advancements in diagnosis and treatment, disparities in lung cancer outcomes persist, particularly in low- and middle-income countries (LMICs), where limited healthcare resources significantly impact early detection and management [3]. In high-income regions such as America and Europe, lung cancer accounted for 13% and 11.8% of all recorded cancers, respectively, in 2021, and constituted one-fifth of cancer-related deaths in the same year [2, 4]. Southern and northern African countries have reported higher prevalence rates of lung cancer compared with Eastern and Western African countries [5]. In sub-Saharan Africa (SSA), the incidence of lung cancer is reportedly lower than in high-income regions, yet this is likely an underestimation due to inadequate diagnostic infrastructure and incomplete cancer registry data [6]. Shifting epidemiological trends, including rising smoking prevalence and increased environmental exposure to carcinogens, underscore the urgent need for context-specific data to inform healthcare planning and policy in the SSA region [1].

Although women are less than half as likely to die from lung cancer as men, the disease has a significant impact on female populations. In 2017, lung cancer surpassed breast cancer as the leading cause of cancer deaths among women in Europe, with 14.6 deaths per 100,000 women [7]. Survival rates remain poor globally, but advancements in personalised treatment and the introduction of targeted therapies have improved outcomes, especially in high-resource settings [8]. For instance, Scandinavian studies have shown significant improvements in 1-year survival among patients with non-squamous cell carcinoma (SCC), particularly in early-stage cases [9, 10]. Factors such as age, gender, performance status, tumour location, histological type and initial treatment modality influence survival outcomes [11, 12].

Molecular profiling has revolutionised lung cancer treatment by enabling personalised therapeutic strategies, including the use of novel targeted and immunotherapies that have significantly improved survival rates [13]. However, in SSA, the implementation of molecular diagnostics and advanced therapies remains limited, and the impact of these advancements on cancer patients’ outcomes is poorly documented [14]. Ghana, like many LMICs, faces multiple challenges in cancer care delivery, including late presentation, lack of robust cancer registry systems and limited access to comprehensive oncological care [15]. Lung cancer represents 3.3% of all cancers in Ghana but accounts for 5.4% of cancer-related deaths [16]. Most patients present with advanced-stage disease, resulting in a poor prognosis. Contributing factors include inadequate access to diagnostic tools, lack of molecular profiling for personalised therapies and limited cancer care resources. Notably, inadequate management and underdiagnosis of lung cancer are alarming issues in the African healthcare setting [5, 17, 18]. Major challenges experienced by lung cancer patients in Ghana include high cost of treatment, inadequate knowledge about cancer, poor health seeking behavior and long distance to access cancer treatment facilities [19].

There are currently three radiotherapy and cancer treatment centres in Ghana, providing critical insights into the prevalence, morbidity and mortality rates of various malignancies, including lung cancer, yet comprehensive data on the management and survival outcomes of lung cancer remain scarce. The study, therefore, aimed to address these knowledge gaps by describing the clinical profiles, management and survival outcomes of patients with lung cancer.

## Methods

### Study design and setting

The research was a quantitative cross-sectional study conducted at one of the largest oncology and radiotherapy centres in SSA. The study site offers unique insights into the challenges and opportunities associated with managing lung cancer in limited-resource settings. The diversity in patient demographics provides a rich sample for understanding the management and survival outcomes of lung cancer in the subregion. The centre is equipped with advanced facilities for cancer diagnosis, staging, and treatment and facilitates access to histopathological and molecular services, critical for lung cancer staging. Also, the centre provides a full spectrum of treatment options for lung cancer, including advanced radiotherapy techniques, targeted and chemotherapy as well as emerging options for immunotherapy.

### Study population

The study population comprised adult patients (≥ 18 years) diagnosed with lung cancer who received treatment between January 01, 2013 and December 31, 2022. Eligibility screening was conducted systematically, whereby all medical records of lung cancer patients were reviewed to confirm a histopathological diagnosis of primary lung malignancy. Patients with incomplete medical records or those whose diagnosis could not be definitively verified were excluded from the study. Patients were recruited regardless of the type of treatment received. Patients with lung metastasis arising from other primary or unknown cancer sites were not included in the study. Also, patients with either synchronous or metachronous lung cancer were excluded from the study.

### Study size and sampling method

A consecutive random sampling technique was used to select eligible participants for the study. Initially, all eligible patients meeting the predefined inclusion criteria were identified. From the consecutively selected pool of participants, a random sample of 118 patients were subsequently selected. Based on Slovin’s formula, a sample size of 115 participants was estimated to be appropriate for the study. This approach ensured that the study captured an accurate representation of lung cancer cases managed at the institution, thereby strengthening the reliability of the reported clinical data and survival outcomes.

### Data collection

Relevant data were extracted from patients’ hospital-based medical records using a standardised data collection tool between 1st and 30th July, 2024. Demographic data recorded included participants’ age, sex and place of residence, whereas clinical data included Eastern Cooperative Oncology Group (ECOG) performance status, family history, body mass index (BMI) and body surface area (BSA). Pathological data recorded included tumour histology, stage at diagnosis based on the 8th edition of the American Joint Committee on Cancer stage classification (AJCC) [20], and histological subtypes. Information regarding molecular biomarkers such as epidermal growth factor receptor (EGFR), anaplastic lymphoma kinase (ALK), Kirsten rat sarcoma viral oncogene homolog (KRAS) and programmed death-ligand (PD-L1) were also recorded if available. Additionally, treatment data were also extracted, including the type, sequence and timing of treatment modalities such as radiotherapy and systemic therapies. Treatment outcome data extracted included the date of diagnosis, last follow-up and survival status (whether deceased or still living).

### Bias

The analysis was conducted with de-identified data to prevent demographic or clinical characteristics from inadvertently influencing the results. This measure aimed to enhance the validity and reliability of the study’s outcomes while addressing potential biases associated with retrospective research.

### Data management and analysis

Data were discretely handled, securely stored and analysed with STATA (version 17) as well as Microsoft Excel for Windows 11. Data were cleaned and coded prior to analyses. Descriptive statistics such as means, standard deviations, percentages and frequency distributions were used to summarise demographic and clinical characteristics. Results were presented in the form of graphs and tables for clarity. Kaplan–Meier survival analyses were used to estimate overall survival (OS) outcomes. Cox proportional hazards models were used to identify prognostic factors associated with survival outcomes.

### Ethical considerations

Ethical approval was obtained from the institutional review board (SBAHS/AA/RAD/11361700/2023-2024). Informed consent was obtained directly from patients who were still alive at the time of the study and from either the next of kin or a legally authorised representative of those who were deceased. Additionally, patients’ medical records were discreetly extracted and analysed anonymously with the removal of all patient-identifying information to protect patients’ privacy and ensure the confidentiality of their information. The study adhered to established ethical standards for retrospective research, ensuring the protection of participants’ rights.

## Results

### Baseline characteristics

The study involved 118 adult patients (≥18 years) comprising 53.4% males and 46.6% females, with a mean age of 59.4 years (± 13), ranging from 24 to 98 years. Notably, 8.5% were younger than 40 years, whereas 48.3% were geriatric (> 60 years) as summarised in Table 1. A considerable majority were married (71.1%), employed (94.9%), urban dwellers (72.1%) and without comorbidities (60.2%). Also, 55.1% had no history of alcohol intake, while 57.6% were nonsmokers. Additionally, 6.8% had a positive family history of malignant neoplasms, whereas 4.2% had been previously treated for pulmonary tuberculosis (PTB).

### Clinical characteristics

The mean BMI was 23.6 kg/m^2^ (± 4.4), ranging from 14.5 to 36.6 kg/m^2^. In all, 20.4% were underweight, whereas 16.1% were overweight and 5% were obese (Table 2). The mean BSA was 1.9 m^2^ (± 1.9), ranging from 1.4 to 2.2 m^2^. Also, 3.4% had BSA < 1.5 m^2^, whereas 5.9% had BSA > 2.0 m^2^. In all, 53.4% had a good performance status of ECOG 0 - 1, whereas 22% had a poor performance status of ECOG 3 - 4. For some patients, the primary tumour was in the right lung (47.5%), whereas 40.6% had left-sided lung cancer, with 11.9% having bilateral lung cancer. Stage IB lung cancer accounted for 5.9% of cases, while 7.6% were stage IIIA. There was a predominance of late-stage diagnoses, with over 75% of patients with stages IVA (48.3%) and IVB (27.1%) lung cancer. There was a stark predominance of non-small cell lung cancer (NSCLC) (94.9%) with small cell lung cancer (SCLC) accounting for only 5.1%.

**Figure 1.**
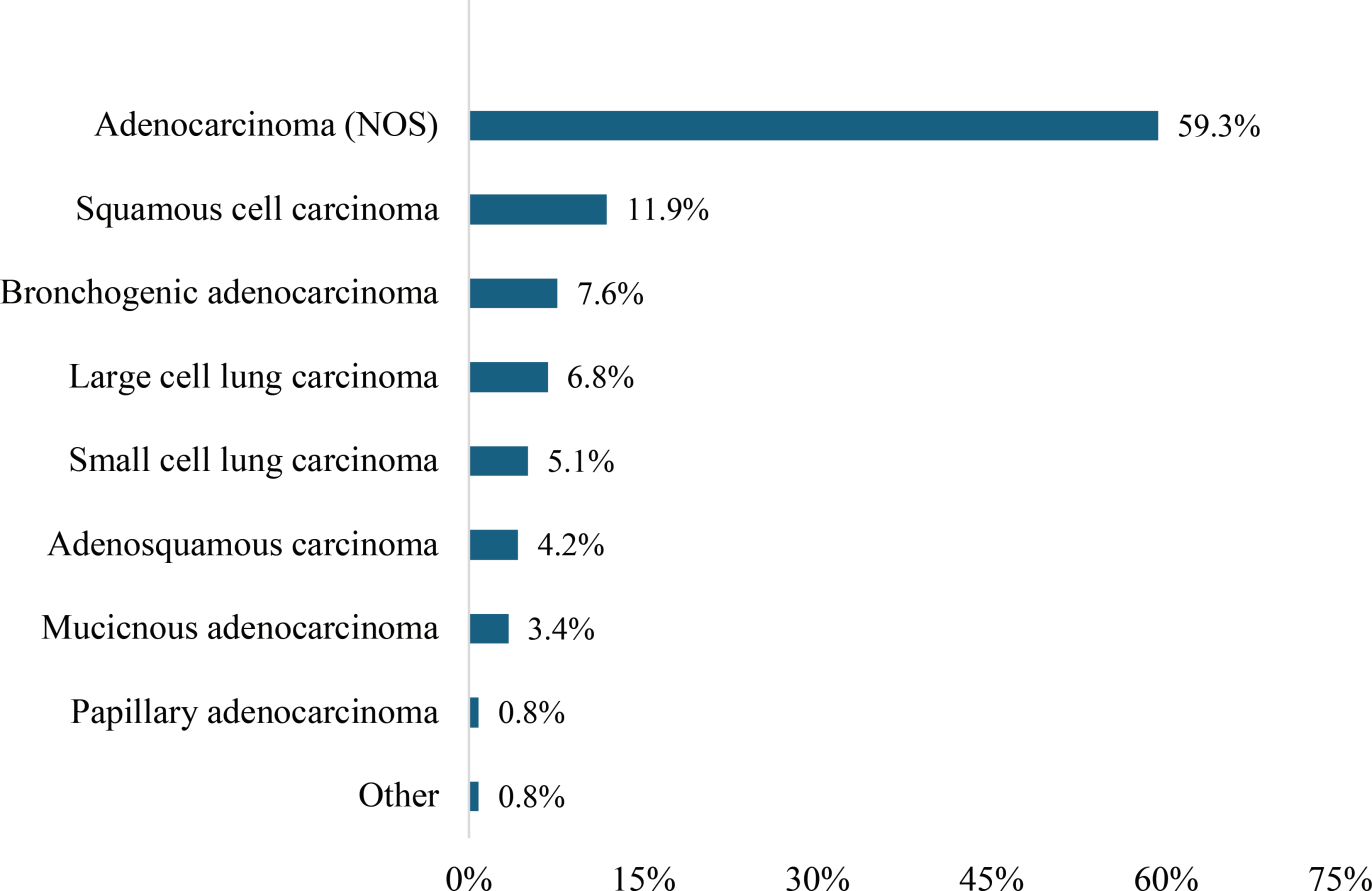
Histological types of lung carcinoma.

Adenocarcinoma (not otherwise specified, NOS) was the most prevalent histological type (59.3%) of NSCLC, whereas SCC accounted for 11.9%. Large cell lung carcinoma constituted 6.8%, whereas adenosquamous carcinoma and mucinous adenocarcinoma accounted for 4.2% and 3.4%, respectively, as illustrated in Figure 1. Papillary adenocarcinoma and other less common subtypes each represented 0.8%.

### Tumour-related characteristics

A few patients had mediastinal (5.1%), hilar (5.9%), axillary (1.7%) and para-aortic (0.8%) lymphadenopathy. Also, a considerable minority had peri-bronchial thickening (1.7%), lung collapse (0.8%), pneumothorax (0.8%) and sub-pleural nodules (2.5%). In all, 57.6% had pleural whereas 2.5% had pericardial effusion. Important sites of distant metastasis were the liver (9.3%), brain (7.6%) and bone 16.9%). There were no statistically significant odds of patients having any of these tumour-related characteristics, as shown by *p*-values >0.05 in Table 3. Data on molecular biomarkers (EGFR, ALK, KRAS and PD-L1) were available for only six patients. Notably, there was a limited presence of EGFR (33.3%) and KRAS mutations (33.3%), as well as ALK rearrangement (16.7%) and PD-L1 expression (16.7%).

### Treatment-related characteristics

Only 24.6% of the patients were treated with curative intent. In all, 65.3% received palliative treatment, whereas 10.1% received best supportive care only (without active antineoplastic treatment). Treatment modalities employed comprised radiotherapy (26.3%), surgery (3.4%) and systemic therapy (72.3%). Systemic therapy included chemotherapy (70.3%), targeted (3.4%) and immunotherapy (1.7%). In all, 5.9% and 17.8% achieved complete and partial response, respectively, whereas 16.1% developed progressive disease after initial treatment (Table 4). The treatment completion rate was 84.9%, with a 21.2% prevalence of treatment-induced toxicity, which accounted for 37.5% of treatment non-completion. Other reasons for the non-completion of recommended treatment were poor ECOG performance status (31.2%), patients’ mortality during treatment (12.5%) and patient’s non-adherence to the prescribed treatment regimen (18.8%).

### Survival outcomes

**Figure 2.**
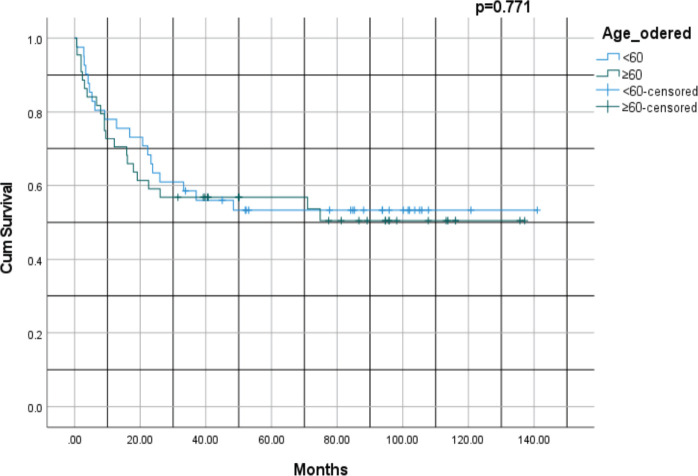
Kaplan–Meier survival analysis of lung cancer by age group: < 60 versus ≥ 60 years.

Figure 2 shows a Kaplan–Meier survival curve comparing two age groups (< 60 versus ≥ 60 years) with a *p*-value of 0.771. For patients < 60 years, the 1-, 3-, 5- and 10-year survival rates were 85%, 65%, 55% and 5%, respectively, whereas for patients ≥ 60 years, survival rates at similar timepoints were 80%, 60%, 5% and 4.5%, respectively. Thus, patients’ age at the time of diagnosis was not a significant factor in survival outcomes for this population.

**Figure 3.**
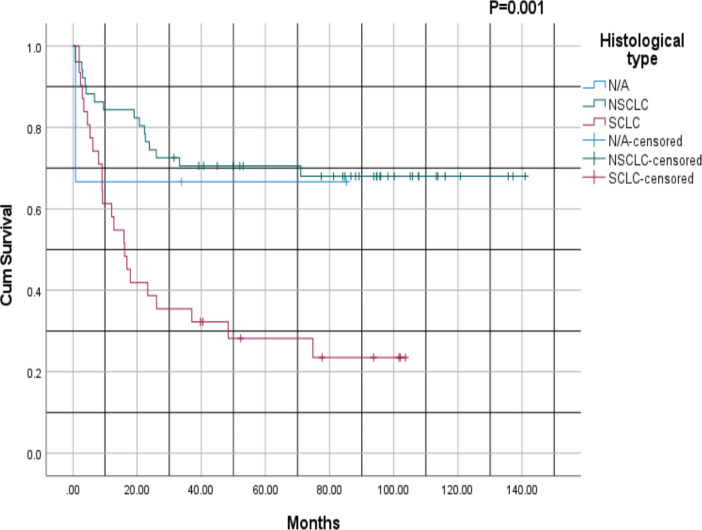
Kaplan–Meier survival analysis based on histological subtype: NSCLC versus SCLC; NSCLC: Non-small cell lung cancer, SCLC: Small cell lung cancer.

Figure 3 compares the survival outcomes of the two main histological subtypes of lung cancer: NSCLC versus SCLC, with a *p*-value of 0.001. For patients with NSCLC, the 1- and 3-year survival rates were 70% and 65%, respectively, whereas for those with SCLC, the survival rates at 1- and 3-years were 45% and 30%, respectively. The NSCLC survival curve plateaus after ~3 years, whereas that for SCLC plateaus after ~4 years. Notably, NSCLC was associated with a better prognosis than SCLC, with higher survival rates across all time points.

**Figure 4.**
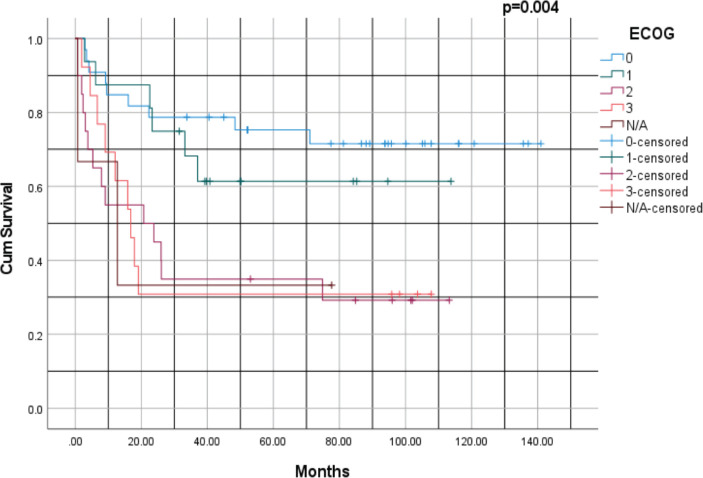
Kaplan–Meier survival analysis of lung cancer based on ECOG performance status. ECOG: Eastern Cooperative Oncology Group.

Figure 4 illustrates the survival rates of patients based on their ECOG performance status with a *p-*value of 0.004. Patients with an ECOG score of 0 had the highest survival rates. At 1 year, the survival rate was ~ 95%, 85% at 3 years, 80% at 5 years and ~75% at 10 years. For patients with a performance status of ECOG 1, the survival rate at 1 year was ~90%, 70% at 3 years, 60% at 5 years and 50% at 10 years. Patients who were ECOG 3 had the poorest survival outcomes. At 1 year, the survival rate was ~40%, 20% at 3 years and 10% at 5 years. Overall, patients with lower ECOG scores (0 and 1) had significantly better survival rates compared to those with higher scores (ECOG 2 and 3).

## Discussion

The study aimed to describe the management and survival outcomes of lung cancer patients at a leading radiotherapy centre in SSA. There were 118 patients (53.4% males and 46.6% females), with a mean age of 59.4 years (± 13). Notably, 48.3% were geriatric (> 60 years). Also, 57.6% were nonsmokers and 6.8% had a positive family history of cancer. In all, 4.2% had a history of treatment for PTB. Over 75% were diagnosed with stages IVA (48.3%) and IVB (27.1%) lung cancer. Most patients were diagnosed with NSCLC (94.9%). Most patients (65.3%) received palliative treatment, while 10.1% received no active antineoplastic treatment at all. Systemic (72.3%) and radiation therapy (26.3%) were the mainstay of treatment. Systemic therapy was mainly chemotherapy (70.3%), with a few patients receiving targeted (3.4%) and immunotherapy 1.7%). There was a high treatment completion rate of 84.9%, although 18.8% were not adherent to the prescribed treatment regimen. Patients <60 years had better OS compared with those ≥60 years, although the difference was not statistically significant (*p* = 0.771). However, patients with NSCLC had statistically significantly better OS than patients with SCLC (*p* = 0.001). Overall, patients with a good ECOG performance status had significantly better survival rates compared to those with a poorer performance status (*p* = 0.004).

The findings of the study provide a comprehensive overview of the socio-demographic, clinical, treatment-related and survival characteristics of 118 patients treated at this centre. These insights highlight significant challenges and opportunities for improving lung cancer management in resource-limited settings, emphasising the need for targeted interventions and systemic improvements. The study population predominantly comprised geriatric patients (48.3%), with a mean age of 59.4 years, aligning with global data that indicate a higher incidence of lung cancer in older populations [3]. The male predominance (53.4%) reflects established gender disparities, often attributed to higher tobacco use and occupational exposures among men [21]. However, the high proportion of non-smokers (57.6%) underscores the importance of non-tobacco-related risk factors, such as environmental pollutants, biomass exposure and genetic predispositions, which are becoming increasingly recognised in SSA [22]. This trend suggests a shifting epidemiology of lung cancer that requires further exploration to inform public health strategies and preventive measures.

The proportion of patients with primary lung cancer with a history of treatment for PTB (4.2%), highlights a potential epidemiological and clinical link between tuberculosis and lung cancer, particularly in regions where tuberculosis remains endemic. The overlap between these two diseases is well-documented, with a systematic review and meta-analysis reporting that individuals with a history of PTB have an increased risk of developing lung cancer compared to those without such a history [23]. The association of PTB with lung cancer could be due to chronic inflammation and lung damage, delayed diagnosis due to similar presentation with symptoms such as night sweats, hemoptysis and unexplained weight loss or coincidental coexistence [24]. Notably, reasons why patients with lung cancer may be treated for PTB include lung cancer misdiagnosis as PTB, true coinfection with PTB or the overlap in radiological findings leading to empirical PTB therapy [23–25]. Clinicians, especially primary caregivers, need to maintain a high index of suspicion for lung cancer in patients with a history of PTB presenting with persistent pulmonary symptoms. Early and accurate differentiation between PTB and lung cancer can be crucial, particularly in regions with high prevalence of tuberculosis.

The predominance of late-stage diagnoses (stages IVA and IVB, accounting for ~ 75%) highlights systemic barriers to early healthcare access and diagnostic services. Limited awareness, inadequate screening programs and infrastructural deficiencies likely contribute to these delays, mirroring patterns observed in other low-resource settings [26]. Late-stage diagnosis complicates treatment, reduces survival outcomes and increases the burden on healthcare systems. The predominance of NSCLC (94.9%), mainly adenocarcinoma (59.3%), is consistent with global trends and suggests that adenocarcinoma is becoming the dominant histological subtype worldwide due to reduced smoking prevalence and improved diagnostic accuracy [27].

Adenocarcinoma, the most common histological subtype (64%), highlights the importance of accurate histological diagnosis and the potential for targeted therapies. Comprehensive molecular profiling, standard in high-resource settings, is essential for identifying actionable mutations and tailoring treatments [28]. However, the limited availability of molecular diagnostic tools in Ghana reflects a significant gap in cancer care infrastructure. Enhanced access to molecular profiling could transform treatment strategies, especially for patients with actionable genetic mutations. Advanced-stage disease was frequently associated with complications such as pleural effusion (34%) and mediastinal lymph node involvement, both of which have significant implications for staging and prognosis. The rarity of liver metastases and bronchial lesions suggests variability in disease presentation but underscores the need for thorough diagnostic evaluations. Treatment response was limited, with only 8% achieving complete or partial responses, reflecting the challenges of managing advanced lung cancer in resource-constrained settings. Improved access to multimodal treatments, including combined chemotherapy, radiotherapy and targeted therapy, could enhance outcomes.

Management strategies for lung cancer were predominantly palliative (65.3%), reflecting the advanced disease stages at presentation. Systemic therapy, primarily chemotherapy (70.3%), was the mainstay of treatment, though access to targeted therapy (3.4%) and immunotherapy (1.7%) was severely limited. This disparity underscores the significant resource constraints faced by healthcare providers in this setting. Advanced oncologic therapies, such as tyrosine kinase and immune checkpoint inhibitors, have revolutionised lung cancer management globally but remain largely inaccessible in SSA due to high costs and limited availability [29]. Radiotherapy, utilised in 26.3% of cases, played a critical role in symptom palliation and local disease control. While treatment completion rates were commendably high (84.9%), non-completion due to treatment-induced toxicity (21.2%) underscores the need for robust supportive care systems. Factors such as poor performance status, logistical challenges and mortality during treatment further highlight the complexities of managing lung cancer in resource-limited settings. Enhanced supportive care, including effective symptom management and nutritional support, could improve treatment tolerance and outcomes.

Survival outcomes were significantly influenced by disease stage, histological subtype and performance status. Kaplan–Meier analysis revealed better survival rates for patients with NSCLC compared to those with SCLC (*p* = 0.001). This finding aligns with established literature documenting the aggressive nature of SCLC, characterised by rapid progression, early metastasis and poor prognosis despite initial responsiveness to therapy [30]. Performance status, as measured by ECOG scores, was a robust predictor of survival. Patients with lower ECOG scores (0 or 1) demonstrated significantly better survival outcomes compared to those with higher scores (*p* = 0.004). This underscores the importance of functional assessment in prognostication and treatment planning. Interestingly, age at diagnosis did not significantly influence survival outcomes (*p* = 0.771), suggesting that other factors, such as stage at presentation, comorbidities and treatment modality, may play more critical roles in determining survival outcomes.

Overall, the findings of the study, highlight the urgent need for systemic improvements in lung cancer care in limited-resource settings. Enhanced early detection and diagnostic capabilities are critical to reducing late-stage presentation. Public health initiatives should focus on increasing awareness of lung cancer symptoms, promoting risk factor mitigation and establishing screening programs for high-risk populations. Additionally, investments in diagnostic infrastructure, such as molecular testing capabilities, are essential for enabling personalised treatment approaches. Improved access to advanced therapies, including targeted and immunotherapies, requires concerted efforts to reduce cost barriers and increase availability. A multidisciplinary approach to patient care, encompassing robust supportive care frameworks and individualised treatment plans, could mitigate treatment-induced toxicities and improve patient outcomes. Population-specific research is also needed to elucidate the unique epidemiological and biological characteristics of lung cancer in SSA, which could inform tailored interventions.

## Limitations

The retrospective design may have introduced selection bias, since data were extracted from existing medical records, which may not have comprehensively captured all patient variables and outcomes. Additionally, the relatively small sample size (118 patients) limits the generalisability of the findings to the broader population of lung cancer patients in SSA. Furthermore, the study was conducted at a single centre, potentially limiting the applicability of findings to other regions with differing healthcare systems and patient demographics. There is a need for prospective, multicentre studies with larger cohorts and more robust data collection systems to validate and expand upon the current findings. Another limitation is the lack of detailed molecular and genetic data, which are increasingly recognised as critical for understanding lung cancer pathophysiology and guiding treatment decisions.

## Conclusion

There was a male predominance among the patients, and a high mean age of 59.4 years, with a significant proportion being geriatric (> 60 years). Also, there were more nonsmokers than smokers (57.6% versus 33.9%), with only 6.8% having a positive family history for cancer. Notably, a small minority had a history of treatment for PTB. Most patients were diagnosed with advanced stages of the disease. Management was essentially palliative (65.3%), with the use of systemic therapy (72.3%). Patients <60 years had better OS compared with those ≥60 years (*p* = 0.771). Similarly, patients with NSCLC had better OS than patients with SCLC (*p* = 0.001). A good ECOG performance status was a predictor of better OS (*p* = 0.004). The study provides valuable insights into the management and survival outcomes of lung cancer patients in resource-limited settings. The predominance of advanced-stage disease, reliance on palliative care and disparities in access to advanced therapies highlight significant challenges faced by healthcare providers in SSA. Addressing these barriers through targeted interventions, infrastructure investments and policy changes could significantly improve lung cancer outcomes in the region. A focus on early detection, comprehensive diagnostics and equitable access to advanced treatments is essential for enhancing survival rates and quality of life for lung cancer patients in this region.

## Conflicts of interest

The authors declare that they have no conflicts of interest.

## Funding

The authors declare that no funds, grants, awards or other support were received either during the conduct of the study or during the preparation of the manuscript.

## Figures and Tables

**Table 1.** Socio-demographic characteristics (*N* = 118).

Characteristics	Variables	Frequency (*n*)	Percentage (%)
Gender	Male	63	53.4
	Female	55	46.6
Age	Mean = 59.4 years (± 13), range = 24–98 years		
	< 40	10	8.5
	40–60	51	43.2
	61–80	54	45.8
	> 80	3	2.5
Marital status	Divorced	12	10.2
	Widowed	8	6.8
	Single	14	11.9
	Married	84	71.1
Employment status	Employed	112	94.9
	Unemployed	4	3.2
	Retired	2	1.9
Residence	Urban	85	72.1
	Rural	33	27.9
Smoking history	Yes	40	33.9
	No	68	57.6
	Not specified	10	8.5
Alcohol intake	Yes	42	35.6
	No	65	55.1
	Not specified	11	9.3
Family history of cancer	Yes	8	6.8
	No	110	93.2
comorbidities	Present	47	39.8
	Absent	71	60.2
Previous treatment for tuberculosis	Yes	5	4.2
	No	113	95.8

**Table 2.** Clinical characteristics (*N* = 118).

Characteristics	Variables	Frequency (*n*)	Percentage (%)
**BMI (kg/m^2^)**			
Mean = 23.6 (± 4.4), range = 14.5–36.6			
Underweight	< 18.5	24	20.4
Normal weight	18.5–24.9	69	58.5
Overweight	25.0–29.9	19	16.1
Obesity cass I (Mild)	30.0–34.9	4	3.3
Obesity class II (Moderate)	35.0–39.9	2	1.7
Obesity class III (Severe)	≥ 40.0	0	-
BSA (m^2^)	Mean = 1.9 (± 1.9), range = 1.4–2.2		
	<1.5	4	3.4
	1.5–2	107	90.7
	> 2	7	5.9
Performance status	ECOG 0	16	13.6
	ECOG 1	47	39.8
	ECOG 2	29	24.6
	ECOG 3	23	19.5
	ECOG 4	3	2.5
Affected lung	Right	56	47.5
	Left	48	40.6
	Bilateral	14	11.9
Clinical stage grouping (AJCC 8th edition) [20]	IB	7	5.9
	IIIA	9	7.6
	IIIB	13	11.1
	IVA	57	48.3
	IVB	32	27.1
Histological subtype	SCLC	6	5.1
	NSCLC	112	94.9

ECOG: Eastern Cooperative Oncology Group, AJCC: American Joint Committee on Cancer, SCLC: Small cell lung cancer, NSCLC: Non-small cell lung cancer

**Table 3.** Tumour-related characteristics (*N* = 118).

Characteristics	Variables	Frequency (n)	Percentage (%)	Odd’s ratio	*p* - value (95% CI)
Mediastinal lymphadenopathy	Absent	112	94.9	3.384	0.195
	Present	6	5.1		
hilar lymphadenopathy	Absent	111	94.1	1.387	0.780
	Present	7	5.9		
axillary lymphadenopathy	Absent	116	98.3	2.230	0.602
	Present	2	1.7		
Para-aortic lymphadenopathy	Absent	117	99.2	1.109	1.000
	Present	1	0.8		
Peri-bronchial thickening	Absent	116	98.3	0.499	1.000
	Present	2	1.7		
Lung collapse	Absent	117	99.2	1.109	1.000
	Present	1	0.8		
Pneumothorax	Absent	117	99.2	2.180	0.347
	Present	1	0.8		
Sub-pleural nodules	Absent	115	97.5	3.364	0.263
	Present	3	2.5		
Pericardial effusion	Absent	115	97.5	3.364	0.263
	Present	3	2.5		
Pleural effusion	Absent	50	42.4	4.462	0.093
	Present	68	57.6		
Liver metastasis	Absent	107	90.7	3.350	0.339
	Present	11	9.3		
Brain metastasis	Absent	109	92.4	3.364	0.263
	Present	9	7.6		
Bone metastasis	Absent	98	83.1	2.698	0.201
	Present	20	16.9		
**Molecular analysis (*n* = 6)**					
EGFR mutation	Positive	2	33.3	-	-
	Negative	4	66.7	-	-
ALK rearrangement	Positive	1	16.7	-	-
	Negative	5	83.3	-	-
KRAS mutation	Positive	2	33.3	-	-
	Negative	4	66.7	-	-
PD-L1 expression	Positive	1	16.7	-	-
	Negative	5	83.3	-	-

EGFR: Epidermal growth factor receptor, ALK: Anaplastic lymphoma kinase, KRAS: Kirsten rat sarcoma viral oncogene homolog, PD-L1: Programmed Death-Ligand

**Table 4.** Treatment-related characteristics.

Clinical characteristics	Variables	Frequency	Percentage (%)
Treatment intent/ approach	Curative	29	24.6
	Palliative	77	65.3
	Best supportive care only	12	10.1
*Treatment modality (*n* = 106)	Radiotherapy	31	26.3
	Surgery (* lobectomy)	4	3.4
	Systemic therapy	86	72.3
**Type of systemic therapy (*n* = 86)	Chemotherapy	83	70.3
	Targeted therapy	4	3.4
	Immunotherapy	2	1.7
Treatment response	Complete response	7	5.9
	Partial response	21	17.8
	Stable disease	39	33.1
	Progressive disease	19	16.1
	Not applicable/ unknown	32	27.1
Treatment-induced toxicity	Yes	25	21.2
	No	68	57.6
	Not applicable/ unknown	25	21.2
Treatment completion (*n* = 106)	Yes	90	84.9
	No	16	15.1
Reasons for non-completion of prescribed treatment (*n* = 16)	Toxicity	6	37.5
	Poor performance status	5	31.2
	Mortality	2	12.5
	Patients’ non-adherence	3	18.8

* Treatment modalities and

** types of systemic therapy utilised were not mutually exclusive. Some patients received multi-modality treatment. Also, systemic treatment administered comprised chemotherapy and/or targeted and/or immunotherapy
